# 

**Published:** 1876-11

**Authors:** 


### Contents of November Number.

### ORIGINAL.

ART. I.—The Rational Treatment of Epilepsy. By Reuben A. Vance, M. D., ........ 123
ART. II.—Sarcoma of the Dura Mater. By E. N. Brush, M. D.,...... 126

### CORRESPONDENCE.

Bergman versus Volker. Reply of Dr. C. C. F. Gay,.. ........ 131

### MISCELLANEOUS.

Salicylate of Soda in Rheumatism,........ 139
The Fascia Lata.—Its use in Standing at Rest, etc.,........ 144
Vaginal Ovariotomy,........ 147
Some Practical Observations in the Differential Diagnosis of Croup and Diphtheria,........ 150
Clinical Conversations on Diseases of the Skin,........ 154

### EDITORIAL.

Bergman vs. Volker,........ 158
Meeting of Alumni Association,........ 160
Books Reviewed:
Transactions of the College of Physicians of Philadelphia,..... 161
Ziemssen's Cyclopædia, Vol. IV........ 162
Books and Pamphlets Received,........ 162

